# Stroke-heart syndrome: Incidence and clinical outcomes of cardiac complications following intracerebral haemorrhage

**DOI:** 10.1177/23969873241264115

**Published:** 2024-07-30

**Authors:** Katie L Hoad, Helen Jones, Gemma Miller, Azmil H Abdul-Rahim, Gregory YH Lip, Benjamin JR Buckley

**Affiliations:** 1 Liverpool Centre for Cardiovascular Science, University of Liverpool, Liverpool John Moores University, and Liverpool Heart & Chest Hospital, Liverpool, UK; 2 Cardiovascular Health Sciences, Research Institute for Sport and Exercise Sciences, Liverpool John Moores University, UK; 3 Department of Cardiovascular and Metabolic Medicine, Institute of Life Course and Medical Sciences, University of Liverpool, Liverpool, UK; 4 Stroke Division, Department Medicine for Older People, Mersey and West Lancashire Teaching Hospitals NHS Trust, Prescot, UK; 5 Department of Clinical Medicine, Aalborg University, Aalborg, Denmark

**Keywords:** Stroke-heart syndrome, intracerebral haemorrhage, arrhythmias, heart failure, outcomes

## Abstract

**Introduction:**

Newly diagnosed cardiovascular complications following an ischaemic stroke, termed stroke-heart syndrome, are common and associated with worse outcomes. Little is known regarding stroke-heart syndrome in relation to intracerebral haemorrhage (ICH). This study aimed to investigate the incidence and 5-year major adverse cardiovascular events (MACE; acute myocardial infarction, ischaemic stroke, all-cause mortality and recurrent ICH) of newly diagnosed cardiovascular complications following incident ICH, using a global federated database.

**Patients and methods:**

A retrospective cohort study was conducted using anonymised electronic medical records. Patients aged ⩾ 18 years with non-traumatic ICH and 5-year follow-up were included. Patients with newly diagnosed cardiovascular complications *within 4-weeks* following the initial ICH were 1:1 propensity score-matched with patients without new-onset cardiovascular complications. Each cardiovascular complications were investigated as a composite stroke-heart syndrome cohort and separately for associated MACE. Cox hazard regression models were used to determine 5-year incidence of MACE.

**Results:**

Before propensity score matching, 171,489 patients with non-traumatic ICH, 15% (*n* = 26,449) experienced ⩾1 newly diagnosed cardiovascular complication within 4 weeks. After matching, patients with ICH and cardiovascular complications were associated with a significantly higher risk of 5-year MACE (HR 1.35 [95% CI 1.32–1.38]), and in each composite compared to matched controls. There was no significant risk of rehospitalisation over 5-year follow-up [HR 0.90 [0.73–1.13]). The risk of MACE was significantly higher in patients with newly diagnosed cardiovascular complications.

**Discussion and conclusions:**

Newly diagnosed cardiovascular complications following ICH (i.e. stroke-heart syndrome) were common and associated with a significantly worsened 5-year prognosis.

## Graphical abstract

**Graphical abstract:**
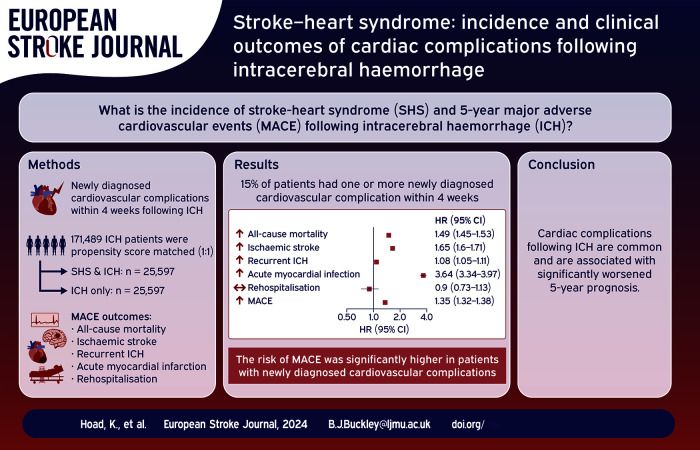

## Introduction

Recent studies have demonstrated that newly diagnosed cardiovascular complications following ischaemic stroke are very common (approximately 20%) and are associated with a poor prognosis, compared to those without newly diagnosed cardiovascular complications following a stroke.^1–3^ The term ‘stroke-heart syndrome’ describes a range of functional, morphological, or biological cardiac changes occurring within the first 30 days following an acute stroke.^2,3^ Clinical manifestations of stroke-heart syndrome include ischaemic heart diseases, heart failure, acute myocardial infarction, atrial and ventricular arrhythmias, and Takotsubo syndrome.^2^ These stroke-induced cardiovascular complications may be caused by inflammation, central autonomic dysfunction, and/or myocardial structural changes.^4,5^

Previous research on stroke-heart syndrome has focussed on ischaemic stroke^2,3,5–7^ with limited attention given to intracerebral haemorrhage (ICH), also termed haemorrhagic stroke. Prior ICH cohorts have generally focussed on pre-existing cardiovascular complications, rather than those with new events following the index ICH presentation. For example, ICH patients with pre-existing cardiovascular complications have an elevated risk of recurrent ICH, ischaemic stroke, and serious vascular events.^8^ Pre-existing atrial fibrillation and heart failure in patients with ICH have been shown to increase the risk of ischaemic stroke and mortality.^8,9^

Some relatively small studies have investigated new cardiovascular events following ICH. For example, a high percentage of patients with ICH experience new cardiac arrhythmias (including severe ventricular arrhythmias and atrial fibrillation) in the early stages following stroke (8%–15%).^10,11^ In a high proportion (15%) these new ECG abnormalities remain up to 2 weeks following ICH.^10^ One retrospective observational study^11^ reported that amongst 1013 patients with ICH, 4.1% (*n* = 39) patients experienced in-hospital cardiovascular complications (i.e. severe ventricular arrhythmia, and heart failure). Furthermore, patients with ICH are at an increased risk of in-hospital acute myocardial infarction and mortality.^11,12^ However, no prior research has investigated the long-term implications of these new-onset cardiovascular complications on major adverse cardiovascular events (MACE).

The aim of this study was therefore to investigate the incidence of newly diagnosed cardiovascular complications following incident ICH, and identify the risk of 5-year major adverse cardiovascular events (MACE), using a large global federated database.

## Methods

This multicentre observational cohort study used anonymised electronic medical records (EMRs) from complete case, anonymised data within TriNetX (https://live.trinetx.com), a global federated health research network with access to electronic medical records (EMRs) from participating healthcare organisations (HCOs), including academic medical centres, specialty physician practices, and community hospitals, predominantly in the United States. As no identifiable information is received in this federated network, research studies using TriNetX do not require ethical approval or patient informed consent.

### Study participants

The network was searched on February 8th, 2024, and identified datasets of included data from 2003 to 2023. Patient records were included with at least 5-years of follow-up from index event (i.e. first record of intracerebral haemorrhage (ICH)). This cohort study adheres to the STROBE (Strengthening the Reporting of Observational Studies in Epidemiology) guidelines.^13^ STROBE checklist can be found in Supplemental Material (Table S1). Patients aged ⩾ 18 years with an incident ICH and a minimum of 5-year follow-up were identified. Only cases with the International Classification of Diseases, 10th Revision, Clinical Modification (ICD-10-CM) code I61 (non-traumatic intracerebral haemorrhage) were included in the analysis, ensuring exclusion of other types of strokes including traumatic haemorrhage. At the time of the search, 73 participating HCOs were included in the network and provided anonymised data. ICH patients who were identified as having a newly diagnosed cardiovascular complication within 4 weeks following an ICH were defined as the exposure (stroke-heart syndrome cohort). The exposure were propensity score-matched in a 1:1 ratio to ICH patients without a new-onset cardiovascular complication (control; ICH only cohort).

### Clinical outcomes

Newly diagnosed cardiovascular complications included heart failure (I50), atrial fibrillation/flutter (AF) (I48), Takotsubo syndrome (I51.81), severe ventricular arrhythmia (i.e. ventricular tachycardia (I47.2) and ventricular fibrillation/flutter (I48)), and ischaemic heart diseases (I20–I25) (Table S2). Each of these cardiac complications were investigated as a composite stroke-heart syndrome cohort (primary analysis) and separately (secondary analyses) for associated 5-year MACE. MACE was defined as the presence of any of the following: recurrent ICH, incident ischaemic stroke, all-cause mortality, acute myocardial infarction. The occurrence of MACE was specified as an event subsequent to the diagnosis of ICH up to 5-years follow-up (Table S3).

### Statistical analysis

Baseline characteristics were compared using χ2 tests or independent-sample t tests. Using logistic regression, the exposure cohort (i.e. stroke-heart syndrome) were 1:1 propensity score matched to control cohort (i.e. ICH only) for age (at index event), sex, ethnicity, hypertensive diseases, diabetes, cerebrovascular diseases (e.g. transient ischaemic attack and sequelae of cerebrovascular disease), pulmonary heart disease/disease of the pulmonary circulation, cardiovascular procedures (including electrocardiography, echocardiography, catheterisation, cardiac devices, and electrophysiological procedures), and cardiovascular medications (including β-blockers, antiarrhythmics, diuretics, antilipemic agents, antianginals, calcium channel blockers, and angiotensin-converting enzyme inhibitors). Comorbidities and cardiovascular care coding are presented in Table S4.

Following propensity score matching, hazard ratios were calculated via Cox hazard regression models with 95% confidence intervals, Kaplan Meier curves and Log Rank *p*-values were also provided for 5-year incidence of MACE comparing ICH patients with newly diagnosed cardiovascular complications to propensity matched controls (without newly diagnosed post-stroke cardiovascular complications). A two-sided *p*-value of less than 0.01 was considered statistically significant to account for multiple testing, reducing the likelihood of Type I error. Sensitivity analyses included excluding all patients with pre-existing cardiovascular and respiratory conditions and patients with multiple cardiac complications following ICH.

## Results

### Clinical characteristics

Before propensity score matching, a total of 171,489 patients (mean age 62.25, SD 19.35; 43.8% female), with ICH were identified from 53 healthcare organisations that met the inclusion criteria with 5-year follow-up (stroke-heart syndrome cohort, *n* = 26,449; ICH cohort, *n* = 145,040). Overall, 15% had one or more newly diagnosed cardiovascular complication within 4-weeks of incident ICH 9% (*n* = 15,413) ischaemic heart disease, 8% (*n* = 14,175) atrial fibrillation/flutter, 6% (9980) heart failure, 2% (*n* = 2608) severe ventricular arrhythmia, and 0.2% (*n* = 409) Takotsubo syndrome.

After propensity score matching, there were 8.7% (*n* = 14,961) patients were identified with ischaemic heart disease, 8.1% (*n* = 13,855) with atrial fibrillation/flutter, 5.6% (*n* = 9622) with heart failure, 1.5% (*n* = 2525) with severe ventricular arrhythmia, and 0.2% (*n* = 409) with Takotsubo syndrome, who were compared to matched controls (ICH without cardiac complications). Overall, cohorts (15%; *n* = 25,597) were deemed well matched for age, sex, ethnicity, comorbidities and cardiovascular procedures/medications, although pulmonary heart disease and diseases of pulmonary circulation remained statistically different between groups after the propensity score matching (Table 1).

**Table 1.** Baseline characteristics *n* (%) of intracerebral haemorrhagic stroke patients with or without cardiovascular complication(s) (i.e. ischaemic heart disease, heart failure, atrial fibrillation/flutter, Takotsubo syndrome and severe ventricular arrhythmia), before and after propensity score matching.

	Before propensity-score matched population			After propensity-score matched population		
	Stroke-heart syndrome cohort (*n* = 26,449)	ICH cohort (*n* = 145,040)	*p-*Value	Stroke-heart syndrome cohort (*n* = 25,597)	ICH cohort (*n* = 25,597)	*p*-Value
Age (yrs) at diagnosis Mean (SD)	68.3 (5.4)	56.2 (23.2)	<0.001	68.3 (15.4)	68.4 (15.6)	0.762
Sex						
Male	13,979 (54.6)	71,642 (52.9)	<0.001	13,968 (54.6)	13,931 (54.4)	0.743
Female	10,815 (42.2)	61,357 (45.3)	<0.001	10,815 (42.3)	10,899 (42.6)	0.453
Ethnicity						
White	16,716 (65.3)	82,225 (60.7)	<0.001	16,705 (65.3)	16,776 (65.5)	0.509
Black or African American	3079 (12.0)	18,659 (13.8)	<0.001	3079 (12.0)	3059 (12.0)	0.786
Asian	969 (3.8)	6191 (4.6)	<0.001	968 (3.8)	980 (3.8)	0.782
Unknown	822 (3.2)	4366 (3.2)	0.908	822 (3.2)	835 (3.3)	0.745
Comorbidities						
Hypertensive diseases	5404 (21.1)	46,635 (34.4)	<0.001	5404 (21.1)	5439 (21.2)	0.705
Diabetes mellitus	2063 (8.1)	19,268 (14.2)	<0.001	2062 (8.1)	2016 (7.9)	0.453
Cerebrovascular diseases	5858 (22.9)	36,082 (26.6)	<0.001	5852 (22.9)	5783 (22.6)	0.467
Chronic kidney disease	963 (3.8)	10,599 (7.8)	<0.001	963 (3.8)	888 (3.5)	0.076
Pulmonary heart disease and diseases of pulmonary circulation	476 (1.9)	6863 (5.1)	<0.001	476 (1.9)	393 (1.5)	0.005
Cardiovascular care						
Procedures	4645 (18.1)	42,094 (31.1)	<0.001	4645 (18.1)	4642 (18.1)	0.973
Medications	7135 (27.9)	58,251 (43.0)	<0.001	7135 (27.9)	7249 (28.3)	0.262

ICH: intracerebral haemorrhage; SD: standard deviation; yrs: years.

*p* < 0.01.

#### Major adverse cardiovascular events and cardiovascular complications

**Figure 1.**
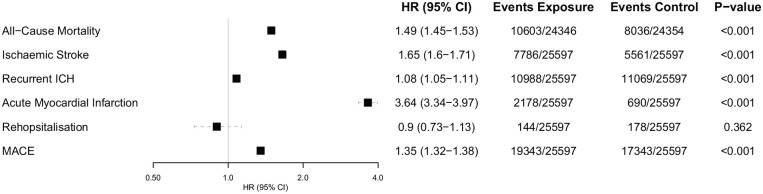
Hazard ratios and 95% confidence intervals for the risk of major adverse cardiovascular events over 5-year follow-up in patients with newly diagnosed cardiovascular complications versus those who were not newly diagnosed with a cardiovascular complications 4-weeks post intracerebral haemorrhagic stroke. CI: confidence interval; ICH: intracerebral haemorrhage; MACE: major adverse cardiovascular events. Hazard ratio (HR), through Cox regression models, reported for propensity-score matched cohort.

Any cardiovascular complication following ICH were associated with significantly higher risk of composite MACE, compared to matched controls without cardiovascular complications (HR 1.35 [95% CI 1.32–1.38]). When investigating each component of MACE individually, there was significantly higher for acute myocardial infarction (HR 3.64 [95% CI 3.34–3.97]), ischaemic stroke (HR 1.65 [95% CI 1.60–1.71]), all-cause mortality (HR 1.49 [95% CI 1.45–1.53]), and recurrent intracerebral haemorrhage (HR 1.08 [95% CI 1.05–1.11]) in patients with ICH and cardiac complications compared to matched controls (Figure 1). There was no significant risk of rehospitalisation over 5 years follow up (HR 0.90 [95% CI 0.73–1.13]).

**Figure 2.**
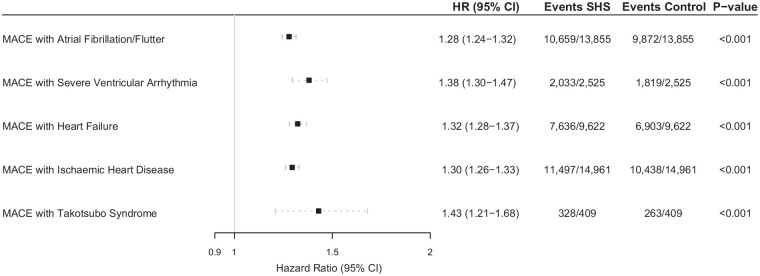
Hazard ratios and 95% confidence intervals for the risk of major adverse cardiovascular events over 5-year follow-up in patients with either atrial fibrillation/flutter, severe ventricular arrhythmias, heart failure, or ischaemic heart diseases versus those who did not have newly diagnosed cardiovascular complications 4-weeks post intracerebral haemorrhagic stroke. CI: confidence interval; ICH: intracerebral haemorrhage; MACE: major adverse cardiovascular events. Hazard ratio (HR), through Cox regression models, reported for propensity-score matched cohort.

When investigating the risk of composite MACE across each cardiovascular complication, there was significantly higher risk for patients with Takotsubo syndrome (HR 1.43 [95% CI 1.21–1.68]), severe ventricular arrhythmia (HR 1.38 [95% CI 1.30–1.47]), heart failure (HR 1.32 [95% CI 1.28–1.37]), ischaemic heart disease (HR 1.30 [95% CI 1.26–1.33]), and atrial fibrillation/flutter (HR 1.28 [95% CI 1.24–1.32]) (Figure 2). In exploratory analysis, multiple cardiovascular complications associated with higher risk of MACE (Figure S1).

#### Individual clinical outcomes

##### Mortality

The risk of 5-year all-cause mortality was significantly higher for patients with ICH and atrial fibrillation/flutter (HR 1.35 [95% CI 1.30–1.40]), severe ventricular arrhythmia (HR 1.81 [95% CI 1.66–1.97]), heart failure (HR 1.52 [95% CI 1.45–1.59]), and ischaemic heart diseases (HR 1.35 [95% CI 1.30–1.40]) compared to matched controls.

##### Recurrent ICH

The 5-year risk of recurrent ICH was significantly higher in patients with heart failure when compared to ICH (HR 1.08 [95% CI 1.03–1.13]). There was no significant difference in risk for recurrent ICH with atrial fibrillation/flutter (HR 1.00 [95% CI 0.97–1.04]), or severe ventricular arrhythmia (HR 1.06 [95% CI 0.97–1.15]), when compared to matched controls.

##### Ischaemic stroke and myocardial infarction

The 5-year risk of ischaemic stroke was significantly higher in all ICH stroke-heart syndrome subgroups: atrial fibrillation/flutter (HR 1.70 [95% CI 1.63–1.78]), heart failure (HR 1.52 [95% CI 1.44–1.60]), severe ventricular arrhythmia (HR 1.44 [95% CI 1.30–1.59]), and ischaemic heart diseases (HR 1.44 [95% CI 1.38–1.51]), compared to matched controls.

The 5-year risk of acute myocardial infarction was significantly higher for patients with ICH and ischaemic heart disease (HR 4.65 [95% CI 4.22–5.13]), heart failure (HR 2.68 [95% CI 2.41–2.98]), severe ventricular arrhythmia (HR 2.64 [95% CI 2.19–3.19]), and atrial fibrillation/flutter (HR 1.72 [95% CI 1.56–1.90]) compared to matched controls. The 5-year risks of acute myocardial infarction had the highest hazard ratio values amongst all MACE outcomes.

##### Takotsubo syndrome

**Figure 3.**
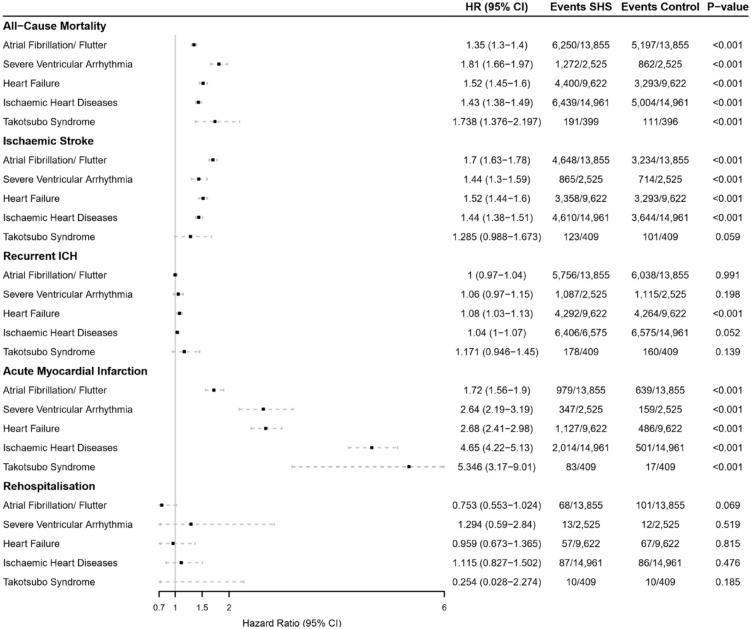
Hazard ratios and 95% confidence intervals for the risk of each major adverse cardiovascular events composites (all-cause mortality, ischaemic stroke, recurrent intracerebral haemorrhage, acute myocardial infarction, rehospitalisation) over 5-year follow-up in patients with either atrial fibrillation/flutter, severe ventricular arrhythmias, heart failure, or ischaemic heart diseases versus those who did not have newly diagnosed cardiovascular complications 4-weeks post intracerebral haemorrhagic stroke. CI: confidence interval; ICH: intracerebral haemorrhage; MACE: major adverse cardiovascular events. Hazard ratio (HR), through Cox regression models, reported for propensity-score matched cohort.

Following ICH, Takotsubo syndrome was associated with significantly higher risk of composite MACE, compared to matched controls without Takotsubo syndrome (HR 1.43 [95% CI 1.21–1.68]) The separated risks of cardiovascular complications to each composite of 5-year MACE can be found in Figure 3.

## Discussion

In this study, our principal findings are (i) newly diagnosed cardiovascular complications within 4 weeks following an ICH were very common (15%; *n* = 26,449), but this varied across different complications, including ischaemic heart disease (9%; *n* = 15,413), followed by atrial fibrillation/flutter (8%; 14,175), heart failure (6%; *n* = 9980), severe ventricular arrhythmia (2%; *n* = 2607), and Takotsubo syndrome (0.2%; n = 409) and (ii) patients with ICH and a newly diagnosed cardiovascular complication were associated with a significantly greater risk of MACE compared to matched controls, over 5 years follow-up from incident ICH.

In recent studies examining MACE outcomes, patients with incident haemorrhagic and ischaemic strokes and newly diagnosed cardiovascular complication were at a significantly higher risk of MACE.^2,14^ Within 5 years, patients with ICH and newly diagnosed cardiovascular complications were at a greater risk of MACE outcomes compared to those without a cardiovascular complication (HR 1.35 [95% CI 1.32–1.38]). When comparing both ischaemic and haemorrhagic stroke cohorts, an overall similar risk of MACE following cardiac complications can be found. Although patients with haemorrhagic stroke may exhibit higher mortality rates, possibly attributed to the severity of stroke.^14^ The risk of MACE culminates within the initial 30 days following ischaemic stroke, likely attributable to stroke-heart syndrome.^15^ In this study, the median occurrence was 13 days for the stroke-heart syndrome cohort and 41 days for ICH only cohort (see Figure S2). Although the risk decreases after 30 days, it remains significant within 90 days and persists 1 year following initial stroke.^15,16^ Two smaller studies have reported the incidence of severe ventricular arrhythmia following ICH ranging from 0.3% to 8% within 30 days of an ICH.^10,11^ When compared to an ischaemic stroke-heart syndrome population and patients following transient ischaemic attack (TIA), the incidence rates of cardiac complications in the current study were largely comparable (2%; *n* = 2607).^2,17^

In the present study, ICH patients with stroke-heart syndrome had a 1.5-fold higher risk of 5-year mortality compared to patients with ICH alone. The greatest risk of 5-year mortality was observed among patients with severe ventricular arrhythmias, closely followed by those with heart failure. In a retrospective study using a Taiwanese insurance database of 608,890 stroke patients (28%; *n* = 173,236 ICH stroke), pre-existing heart failure was associated with an increased risk of post-discharge mortality (OR 2.59 [95% CI 2.07–3.26]) compared to those without pre-existing heart failure.^18^ Although the present study specifically focussed on cardiac complications following ICH, these findings suggest that patients with ICH and heart failure are associated with a higher risk of mortality, irrespective of whether heart failure develops before or after ICH.

In cases of ischaemic stroke, cardiac arrhythmias or ventricular repolarisation changes are the leading cardiac cause of mortality following a stroke.^19^ Specifically, patients with ischaemic stroke-heart syndrome had a twofold higher risk of 5-year mortality, particularly when the cardiac complication was a severe ventricular arrhythmia.^2,4^ The current study shows that ICH patients with newly diagnosed cardiovascular complications had a fourfold greater risk of 5-year acute myocardial infarction compared to matched controls (ICH without cardiac complications). This is similar to previous work in an ischaemic stroke cohort where those newly diagnosed with ischaemic heart disease were at high risk of a future acute myocardial infarction.^2^ Although no known prior work has investigated long-term outcomes of newly diagnosed ischaemic heart disease following ICH, these findings align with previous research on individuals with pre-existing ischaemic heart disease. Specifically, a 3.5% higher risk of acute myocardial infarction was seen at 10-year follow-up in patients with ICH and pre-existing ischaemic heart disease.^18^ Also, Sposato et al.^5^ found that stroke patients with subclinical ischaemic heart diseases or a history of acute myocardial infarction were associated with a heightened risk of future acute myocardial infarction due to stroke-induced accelerated coronary artery atherosclerosis, further highlighting the vulnerability of individuals with ischaemic heart disease to subsequent myocardial infarction following stroke.

Heart failure was associated with a significantly higher risk of 5-year recurrent ICH (HR 1.08). Although the reason(s) for this is unclear, this is similar to ICH patients with pre-existing heart failure, who have a 1.8 times higher 3-year risk of recurrent ICH compared to ICH patients without pre-existing heart failure.^9^ Potential explanations for this may be decompensation, use of anti-thrombotic treatments, and type of ICH (e.g. lobar ICH which is an independent risk factor for rebleeding).^19,20^ Indeed, ICH in relation to AF presents even greater uncertainty especially in relation to whether (and when) thromboprophylaxis should be initiated.^21,22^

Stroke-heart syndrome in patients with ICH did not associate with a higher risk of 5-year rehospitalisation compared to matched controls. It is likely that although rehospitalisation rates did not significantly differ, the cause of rehospitalisation did. It seems probable that for patients with stroke-heart syndrome, rehospitalisation was more likely due to a severe MACE, as denoted by our primary findings, compared to patients with ICH only. Further, rates of rehospitalisation were lower than other MACE outcomes, possibly limiting precision. However, direct measurement of cause of hospitalisation was not possible in this study and warrants future investigation.

The present study highlights the need for a more holistic and integrated care approach to post-stroke management to reduce the cardiovascular risks associated with this high-risk population,^23^ now advocated by a European Society of Cardiology position paper.^24^

### Limitations

Information concerning the severity and location of ICH was unavailable. The data available in TriNetX might originate from specific HCO’s and regions, potentially introducing biases into the dataset. The cohort examined in the current study spans over 20 years, and it is possible that the time window may include differences in stroke management, health record collection, and the impact of COVID 19 pandemic. Moreover, this study does not include acute cardiac changes of stroke-heart syndrome such as cardiac biomarkers (e.g. high sensitivity troponin or NT-proBNP). Instead, it focuses on newly diagnosed, overt clinical cardiovascular complications, as previously reported.^3^ Ultimately, the determination of whether a cardiovascular complication is the result of an ICH event or pre-existed prior to the stroke (potentially exacerbated by ICH), and is subsequently diagnosed due to thorough clinical work up, remains uncertain. Prospective observational studies may be able to explore this concept further, such as in the Liverpool Heart & Brain Project.^25^ Nonetheless, despite these limitations, the clinical importance of newly diagnosed cardiovascular complications following ICH remains.

## Conclusion

Newly diagnosed cardiovascular complications following ICH (i.e. stroke-heart syndrome) were common and associated with a significantly worsened 5-year prognosis. Findings underscore the importance of implementing preventive cardiology measures for these patients and the need for further research in this under studied area.

## Supplemental Material

sj-docx-1-eso-10.1177_23969873241264115 – Supplemental material for Stroke-heart syndrome: Incidence and clinical outcomes of cardiac complications following intracerebral haemorrhage Supplemental material, sj-docx-1-eso-10.1177_23969873241264115 for Stroke-heart syndrome: Incidence and clinical outcomes of cardiac complications following intracerebral haemorrhage by Katie L Hoad, Helen Jones, Gemma Miller, Azmil H Abdul-Rahim, Gregory YH Lip and Benjamin JR Buckley in European Stroke Journal
