# Supplementary material for: Stroke-heart syndrome: Incidence and clinical outcomes of cardiac complications following intracerebral haemorrhage
Source: Eur Stroke J. 2024 Jul 30;10(1):100–7. doi: 10.1177/23969873241264115 (PMC11569547; doi:10.1177/23969873241264115)
Supplement: sj-docx-1-eso-10.1177_23969873241264115 – Supplemental material for Stroke-heart syndrome: Incidence and clinical outcomes of cardiac complications following intracerebral haemorrhage [file sj-docx-1-eso-10.1177_23969873241264115.docx]

## Supplementary Material

**Table S1.** Strengthening the reporting of observational studies in epidemiology (STROBE) statement. Cohort studies’ checklist of included items in report.

|  | Item Number | Recommendations | Page/section  information can  be found |
| --- | --- | --- | --- |
| Title and abstract | 1 | (a) Indicate the study’s design with a commonly used term in the title or the abstract | Page 2; abstract |
|  |  | (b) Provide in the abstract an informative and balanced summary of what was done and what was found | Page 2-3; abstract |
| Introduction | | | |
| Background/ rationale | 2 | Explain the scientific background and rationale for the investigation being reported | Page 4-5;  introduction |
| Objectives | 3 | State specific objectives, including any prespecified hypotheses | Page 5; end of introduction |
| Methods | | | |
| Study design | 4 | Present key elements of study design early in the paper | Page 5-6; methods |
| Setting | 5 | Describe the setting, locations, and relevant dates, including periods of recruitment, exposure, follow-up, and data collection | Page 5-6; methods |
| Participants | 6 | (a) Give the eligibility criteria, and the sources and methods of selection of participants. Describe methods of follow-up | Page 5-6; methods |
|  |  | (b) For matched studies, give matching criteria and number of exposed and unexposed | Page 6; methods |
| Variable | 7 | Clearly define all outcomes, exposures, predictors, potential confounders, and effect modifiers. Give diagnostic criteria, if applicable | Page 6; methods |
| Data sources/ measurements | 8* | For each variable of interest, give sources of data and details of methods of assessment (measurement). Describe comparability of assessment methods if there is more than one group | Page 6; methods |
| Bias | 9 | Describe any efforts to address potential sources of bias | N/a |
| Study size | 10 | Explain how the study size was arrived at | N/a |
| Quantitative variables | 11 | Explain how quantitative variables were handled in the analyses. If applicable, describe which groupings were chosen and why | Page 5+6; methods |
| Statistical methods | 12 | (a) Describe all statistical methods, including those used to control for confounding | Page 7; methods |
|  |  | (b) Describe any methods used to examine subgroups and interactions | N/a |
|  |  | (c) Explain how missing data were addressed | N/a |
|  |  | (d) If applicable, explain how loss to follow-up was addressed | N/a |
|  |  | (e) Describe any sensitivity analyses | Page 7; methods |
| Results | | | |
| Participants | 13* | (a) Report numbers of individuals at each stage of study—e.g. numbers potentially eligible, examined for eligibility, confirmed eligible, included in the study, completing follow-up, and analysed | Page 8; results |
|  |  | (b) Give reasons for non-participation at each stage | N/a |
|  |  | (c) Consider use of a flow diagram | N/a |
| Descriptive data | 14* | (a) Give characteristics of study participants (e.g. demographic, clinical, social) and information on exposures and potential | Page 8; results  + Table 1 + Table S1 |
|  |  | (b) Indicate number of participants with missing data for each variable of interest | N/a |
|  |  | (c) Summarise follow-up time (e.g., average and total amount) | Page Figure S2 |
| Outcome data | 15* | Report numbers of outcome events or summary measures over time | Page 8-10; results  + Figure 1-3 |
| Main results | 16 | a) Give unadjusted estimates and, if applicable, confounder-adjusted estimates and their precision (e.g., 95% confidence interval). Make clear which confounders were adjusted for and why they were included | Page 8-10; results  + Table 1 |
|  |  | (b) Report category boundaries when continuous variables were categorized | N/a |
|  |  | (c) If relevant, consider translating estimates of relative risk into absolute risk for a meaningful time period | Page 8-10; results |
| Other analyses | 17 | Report other analyses done—e.g. analyses of subgroups and interactions, and sensitivity analyses | Figure S1 |
| Discussion | | | |
| Key results | 18 | Summarise key results with reference to study objectives | Page 11 |
| Limitations | 19 | Discuss limitations of the study, taking into account sources of potential bias or imprecision. Discuss both direction and magnitude of any potential bias | Page 14; limitations |
| Interpretation | 20 | Give a cautious overall interpretation of results considering objectives, limitations, multiplicity of analyses, results from similar studies, and other relevant evidence | Page 14; conclusions |
| Generalisability | 21 | Discuss the generalisability (external validity) of the study results | Page 14; conclusions |
| Other information | | | |
| Funding | 22 | Give the source of funding and the role of the funders for the present study and, if applicable, for the original study on which the present article is based | Disclosures |

*Give information separately for exposed and unexposed groups in cohort studies.

**Note:** An Explanation and Elaboration article discusses each checklist item and gives methodological background and published examples of transparent reporting. The STROBE checklist is best used in conjunction with this article (freely available on the Web sites of PLoS Medicine at http://www.plosmedicine.org/, Annals of Internal Medicine at http://www.annals.org/, and Epidemiology at http://www.epidem.com/). Information on the STROBE Initiative is available at http://www.strobe-statement.org.

**Table S2.** International Classification of Diseases 10^th^ Revision (ICD-10-CM) codes for cardiovascular complications diagnosed 4-weeks following an intracerebral haemorrhagic stroke.

| Cardiovascular complication of stroke-heart syndrome | | ICD-10-CM codes | | |
| --- | --- | --- | --- | --- |
| Heart failure | | I50 | | |
| Ventricular tachycardia | | I47.2 | | |
| Ventricular fibrillation and flutter | | I49.0 | | |
| Takotsubo syndrome | | I51.81 | | |
| Ischaemic heart diseases | | I20-I25 | | |
|  | Unstable angina |  | I20 |  |
|  | Acute myocardial infarction |  | I21 |  |
|  | Subsequent ST elevation and non-ST elevation myocardial infarction |  | I22 |  |
|  | Certain current complications following ST elevation (STEMI) and non-ST elevation (NSTEMI) myocardial infarction |  | I23 |  |
|  | Other acute ischaemic heart diseases |  | I24 |  |
|  | Chronic ischaemic heart diseases |  | I25 |  |
| Atrial fibrillation and flutter | | I48 | | |

**Table S3.** International Classification of Diseases 10^th^ Revision codes for 5-year major cardiovascular adverse events.

| **Cardiovascular complication of stroke-heart syndrome** | **ICD-10-CM codes (label)** |
| --- | --- |
| Recurrent intracerebral haemorrhage | I61 (nontraumatic intracerebral haemorrhage) |
| Ischaemic stroke | I63 (cerebral infarction) |
| All-cause mortality | Deceased |
| Acute myocardial infarction | I21 (acute myocardial infarction) |
| Hospitalisation | [SNOMED] 32485007 (hospital admission) |

ICD-10-CM, International Classification of Diseases 10^th^ Revision; MACE, major cardiovascular adverse events; SNOMED, systematized nomenclature of medicine clinical terms

**Table S4.** Baseline characteristics (including pre-stroke comorbidities and cardiovascular care) and coding.

| **Baseline characteristic** | **Code** |
| --- | --- |
| Hypertension | I10-I16 |
| Cerebrovascular disease | I60-I69 |
| Diabetes | E08-E13 |
| Pulmonary disease/disease of the pulmonary circulation | I26-I28 |
| Chronic kidney disease | N18 |
| Cardiovascular care | Procedures (CPT 1012974) |
|  | Medications (VA CV000) |

**Table S5**. Baseline characteristics n (%) of intracerebral haemorrhagic stroke patients with or without atrial fibrillation/flutter before and after propensity score matching.

|  | | Before Propensity-Score Matched Population | | | After Propensity-Score Matched Population | | |
| --- | --- | --- | --- | --- | --- | --- | --- |
|  |  | Stroke-heart syndrome cohort  (*n = 14,175*) | ICH cohort  (*n = 162,216*) | *p* value | Stroke-heart syndrome cohort  (*n = 13,855*) | ICH cohort  (*n = 13,855*) | *p* value |
| Age (yrs) at diagnosis  Mean (SD) | | 72.9 (2.7) | 57.2 (22.7) | <0.001 | 72.9 (12.7) | 72.9 (12.8) | 0.855 |
| Sex | |  |  |  |  |  |  |
|  | Male | 7,434 (53.7) | 80,868 (53.2) | 0.330 | 7,434 (53.7) | 7,414 (53.5) | 0.810 |
|  | Female | 5,907 (42.6) | 68.151 (44.9) | <0.001 | 5,907 (42.6) | 5,947 (42.9) | 0.627 |
| Ethnicity | |  |  |  |  |  |  |
|  | White | 9,601 (69.3) | 92,386 (60.8) | <0.001 | 9,600 (69.3) | 9,653 (69.7) | 0.489 |
|  | Black or African American | 1,188 (8.6) | 21,144 (13.9) | <0.001 | 1,188 (8.6) | 1,184 (8.6) | 0.898 |
|  | Asian | 574 (4.1) | 6,776 (4.5) | 0.083 | 574 (4.1) | 549 (4.0) | 0.446 |
|  | Unknown | 368 (2.7) | 368 (3.2) | <0.001 | 368 (2.7) | 363 (2.6) | 0.851 |
| Comorbidities | |  |  |  |  |  |  |
|  | Hypertensive Diseases | 3,421 (24.7) | 51,538 (33.9) | <0.001 | 3,421 (24.7) | 3,433 (24.8) | 0.867 |
|  | Diabetes Mellitus | 1,440 (10.4) | 21,470 (14.1) | <0.001 | 1,440 (10.4) | 1,397 (10.1) | 0.394 |
|  | Cerebrovascular Diseases | 3,183 (23.0) | 38,992 (25.7) | <0.001 | 3,182 (23.0) | 3,123 (22.5) | 0.398 |
|  | Chronic Kidney Disease | 756 (5.5) | 11,824 (7.8) | <0.001 | 756 (5.5) | 718 (5.2) | 0.309 |
|  | Pulmonary Heart Disease and Diseases of Pulmonary Circulation | 444 (3.2) | 7,548 (5.0) | <0.001 | 444 (3.2) | 379 (2.7) | 0.021 |
| Cardiovascular Care | |  |  |  |  |  |  |
|  | Procedures | 2,912 (21.0) | 46,277 (30.5) | <0.001 | 2,912 (21.0) | 2,910 (21.0) | 0.976 |
|  | Medications | 4,233 (30.5) | 63,996 (42.1) | <0.001 | 4,233 (30.6) | 4,260 (30.7) | 0.725 |

ICH, intracerebral haemorrhage; SD, standard deviation; yrs, years. p < 0.01

**Table S6.** Baseline characteristics n (%) of intracerebral haemorrhagic stroke patients with or without severe ventricular arrhythmia before and after propensity score matching.

|  | | Before Propensity-Score Matched Population | | | After Propensity-Score Matched Population | | |
| --- | --- | --- | --- | --- | --- | --- | --- |
|  |  | Stroke-heart syndrome cohort  (*n = 2,608*) | ICH cohort  (*n = 173,734*) | *p* value | Stroke-heart syndrome cohort  (*n = 2,525*) | ICH cohort  (*n = 2,525*) | *p* value |
| Age (yrs) at diagnosis  Mean (SD) | | 64.2 (17.3) | 58.4 (22.6) | <0.001 | 64.2 (17.3) | 64.7 (17.0) | 0.296 |
| Sex | |  |  |  |  |  |  |
|  | Male | 1,504 (59.6) | 86,765 (53.2) | <0.001 | 1,504 (59.6) | 1,516 (60.0) | 0.731 |
|  | Female | 921 (36.5) | 73,122 (44.8) | <0.001 | 921 (36.5) | 915 (36.2) | 0.861 |
| Ethnicity | |  |  |  |  |  |  |
|  | White | 1,500 (59.4) | 100,453 (61.5) | 0.029 | 1,500 (59.4) | 1,515 (60.0) | 0.667 |
|  | Black or African American | 409 (16.2) | 21,916 (13.4) | <0.001 | 409 (16.2) | 418 (16.6) | 0.732 |
|  | Asian | 88 (3.5) | 7,262 (4.4) | 0.020 | 88 (3.5) | 88 (3.5) | 1.000 |
|  | Unknown | 70 (2.8) | 5,192 (3.2) | 0.245 | 70 (2.8) | 71 (2.8) | 0.932 |
| Comorbidities | |  |  |  |  |  |  |
|  | Hypertensive Diseases | 1,100 (43.6) | 53,471 (32.8) | <0.001 | 1,100 (43.6) | 1,091 (43.2) | 0.798 |
|  | Diabetes Mellitus | 493 (19.5) | 22,319 (13.7) | <0.001 | 493 (19.5) | 503 (19.9) | 0.724 |
|  | Cerebrovascular Diseases | 873 (34.6) | 40,164 (24.6) | <0.001 | 873 (34.6) | 875 (34.7) | 0.953 |
|  | Chronic Kidney Disease | 293 (11.6) | 12,225 (7.5) | <0.001 | 293 (11.6) | 285 (11.3) | 0.724 |
|  | Pulmonary Heart Disease and Diseases of Pulmonary Circulation | 239 (9.5) | 7,690 (4.7) | <0.001 | 239 (9.5) | 200 (7.9) | 0.051 |
| Cardiovascular Care | |  |  |  |  |  |  |
|  | Procedures | 1,009 (40.0) | 47,787 (29.3) | <0.001 | 1,009 (40.0) | 1,005 (39.8) | 0.908 |
|  | Medications | 1,238 (49.0) | 66,508 (40.7) | <0.001 | 1,238 (49.0) | 1,222 (48.4) | 0.652 |

ICH, intracerebral haemorrhage; SD, standard deviation; yrs, years. p < 0.01

**Table S7.** Baseline characteristics n (%) of intracerebral haemorrhagic stroke patients with or without heart failure before and after propensity score matching.

|  | | Before Propensity-Score Matched Population | | | After Propensity-Score Matched Population | | |
| --- | --- | --- | --- | --- | --- | --- | --- |
|  |  | Stroke-heart syndrome cohort  (*n = 9,980*) | ICH cohort  (*n = 166,411*) | *p* value | Stroke-heart syndrome cohort  (*n = 9,622*) | ICH cohort  (*n = 9,622*) | *p* value |
| Age (yrs) at diagnosis  Mean (SD) | | 67.0 (16.8) | 58.0 (22.7) | <0.001 | 67.0 (16.8) | 67.0 (16.8) | 0.698 |
| Sex | |  |  |  |  |  |  |
|  | Male | 5,175 (53.8) | 83,127 (53.2) | 0.290 | 5,175 (53.8) | 5,219 (54.2) | 0.525 |
|  | Female | 4,106 (42.7) | 69,954 (44.8) | <0.001 | 4,106 (42.7) | 4,101 (42.6) | 0.942 |
| Ethnicity | |  |  |  |  |  |  |
|  | White | 5,891 (61.2) | 96,097 (61.5) | 0.544 | 5,891 (61.2) | 5,923 (61.6) | 0.636 |
|  | Black or African American | 1,396 (14.5) | 20,936 (13.4) | 0.002 | 1,396 (14.5) | 1,424 (14.8) | 0.568 |
|  | Asian | 345 (3.6) | 7,005 (4.5) | <0.001 | 345 (3.6) | 346 (3.6) | 0.969 |
|  | Unknown | 277 (2.9) | 4,988 (3.2) | 0.087 | 277 (2.9) | 278 (2.9) | 0.966 |
| Comorbidities | |  |  |  |  |  |  |
|  | Hypertensive Diseases | 3,537 (36.8) | 51,716 (33.1) | <0.001 | 3,537 (36.8) | 3,544 (36.8) | 0.917 |
|  | Diabetes Mellitus | 1,575 (16.4) | 21,468 (13.7) | <0.001 | 1,575 (16.4) | 1,538 (16.0) | 0.481 |
|  | Cerebrovascular Diseases | 3,306 (34.4) | 39,106 (25.0) | <0.001 | 3,306 (34.4) | 3,248 (33.8) | 0.386 |
|  | Chronic Kidney Disease | 828 (8.6) | 11,845 (7.6) | <0.001 | 828 (8.6) | 786 (8.2) | 0.275 |
|  | Pulmonary Heart Disease and Diseases of Pulmonary Circulation | 460 (4.8) | 7,587 (4.9) | 0.731 | 460 (4.8) | 377 (3.9) | 0.003 |
| Cardiovascular Care | |  |  |  |  |  |  |
|  | Procedures | 3,084 (32.0) | 46,374 (29.7) | <0.001 | 3,084 (32.1) | 3,092 (32.1) | 0.902 |
|  | Medications | 3,865 (40.2) | 64,521 (41.3) | 0.027 | 3,865 (40.2) | 3,843 (39.9) | 0.746 |

ICH, intracerebral haemorrhage; SD, standard deviation; yrs, years. p < 0.01

**Table S8.** Baseline characteristics n (%) of intracerebral haemorrhagic stroke patients with or without ischaemic heart diseases before and after propensity score matching.

|  | | Before Propensity-Score Matched Population | | | After Propensity-Score Matched Population | | |
| --- | --- | --- | --- | --- | --- | --- | --- |
|  |  | Stroke-heart syndrome cohort  (*n = 15,413*) | ICH cohort  (*n = 160,978*) | *p* value | Stroke-heart syndrome cohort  (*n = 14,961*) | ICH cohort  (*n = 14,961*) | *p* value |
| Age (yrs) at diagnosis  Mean (SD) | | 68.7 (14.0) | 57.5 (22.9) | <0.001 | 68.7 (14.0) | 68.8 (14.3) | 0.923 |
| Sex | |  |  |  |  |  |  |
|  | Male | 8,691 (58.1) | 79,611 (52.8) | <0.001 | 8,691 (58.1) | 8,693 (58.1) | 0.981 |
|  | Female | 5,780 (38.6) | 68,279 (45.3) | <0.001 | 5,780 (38.6) | 5,802 (38.8) | 0.794 |
| Ethnicity | |  |  |  |  |  |  |
|  | White | 9,802 (65.5) | 92,185 (61.1) | <0.001 | 9,802 (65.5) | 9,874 (66.0) | 0.380 |
|  | Black or African American | 1,869 (12.5) | 20,464 (13.6) | <0.001 | 1,869 (12.5) | 1,844 (12.3) | 0.661 |
|  | Asian | 504 (3.4) | 6,846 (4.5) | <0.001 | 504 (3.4) | 516 (3.4) | 0.702 |
|  | Unknown | 480 (3.2) | 4,785 (3.2) | 0.809 | 480 (3.2) | 475 (3.2) | 0.869 |
| Comorbidities | |  |  |  |  |  |  |
|  | Hypertensive Diseases | 3,752 (25.1) | 51,522 (34.2) | <0.001 | 3,752 (25.1) | 3,732 (24.9) | 0.789 |
|  | Diabetes Mellitus | 1,568 (10.5) | 21,464 (14.2) | <0.001 | 1,568 (10.5) | 1,528 (10.2) | 0.448 |
|  | Cerebrovascular Diseases | 3,668 (24.5) | 38,922 (25.8) | 0.001 | 3,668 (24.5) | 3,563 (23.8) | 0.156 |
|  | Chronic Kidney Disease | 820 (5.5) | 11,826 (7.8) | <0.001 | 820 (5.5) | 707 (4.7) | 0.003 |
|  | Pulmonary Heart Disease and Diseases of Pulmonary Circulation | 438 (2.9) | 7,582 (5.0) | <0.001 | 438 (2.9) | 368 (2.5) | 0.012 |
| Cardiovascular Care | |  |  |  |  |  |  |
|  | Procedures | 3,179 (21.2) | 46,219 (30.6) | <0.001 | 3,179 (21.2) | 3,122 (20.9) | 0.419 |
|  | Medications | 4,635 (31.0) | 63,814 (42.3) | <0.001 | 4,635 (31.0) | 4,694 (31.4) | 0.462 |

ICH, intracerebral haemorrhage; SD, standard deviation; yrs, years. p < 0.01

**Table S9.** Baseline characteristics n (%) of intracerebral haemorrhagic stroke patients with or without Takotsubo syndrome before and after propensity score matching.

|  | | Before Propensity-Score Matched Population | | | After Propensity-Score Matched Population | | |
| --- | --- | --- | --- | --- | --- | --- | --- |
|  |  | Stroke-heart syndrome cohort  (*n = 409)* | ICH cohort  (*n = 289,599*) | *p* value | Stroke-heart syndrome cohort  (*n = 409*) | ICH cohort  (*n = 409*) | *p* value |
| Age (yrs) at diagnosis  Mean (SD) | | 60.5 (16.2) | 59.5 (22.1) | 0.360 | 60.5 (16.2) | 59.6 (17.2) | 0.434 |
| Sex | |  |  |  |  |  |  |
|  | Male | 115 (28.1) | 148,780 (53.3) | <0.001 | 115 (28.1) | 116 (28.4) | 0.938 |
|  | Female | 289 (70.7) | 122,711 (44.0) | <0.001 | 289 (70.7) | 288 (70.4) | 0.939 |
| Ethnicity | |  |  |  |  |  |  |
|  | White | 278 (68.0) | 168,112 (60.3) | 0.001 | 278 (68.0) | 284 (69.4) | 0.651 |
|  | Black or African American | 51 (12.5) | 38,179 (13.7) | 0.475 | 51 (12.5) | 48 (11.7) | 0.748 |
|  | Asian | 12 (2.9) | 12,576 (4.5) | 0.125 | 12 (2.9) | 14 (3.4) | 0.690 |
|  | Unknown | 18 (4.4) | 9,598 (3.4) | 0.287 | 18 (4.4) | 19 (4.6) | 0.866 |
| Comorbidities | |  |  |  |  |  |  |
|  | Hypertensive Diseases | 109 (26.7) | 102,257 (36.6) | <0.001 | 109 (26.7) | 107 (26.2) | 0.874 |
|  | Diabetes Mellitus | 48 (11.7) | 43,727 (15.7) | 0.029 | 48 (11.7) | 44 (10.8) | 0.658 |
|  | Cerebrovascular Diseases | 96 (23.5) | 70,725 (25.3) | 0.383 | 96 (23.5) | 97 (23.7) | 0.934 |
|  | Chronic Kidney Disease | 26 (6.4) | 26,338 (9.4) | 0.033 | 26 (6.4) | 23 (5.6) | 0.658 |
|  | Pulmonary Heart Disease and Diseases of Pulmonary Circulation | 22 (5.4) | 15,503 (5.6) | 0.876 | 22 (5.4) | 18 (4.4) | 0.517 |
| Cardiovascular Care | |  |  |  |  |  |  |
|  | Procedures | 105 (25.7) | 92,553 (33.2) | 0.001 | 105 (25.7) | 103 (25.2) | 0.872 |
|  | Medications | 147 (35.9) | 129.998 (46.6) | <0.001 | 147 (35.9) | 149 (36.4) | 0.884 |

ICH, intracerebral haemorrhage; SD, standard deviation; yrs, years. p < 0.01


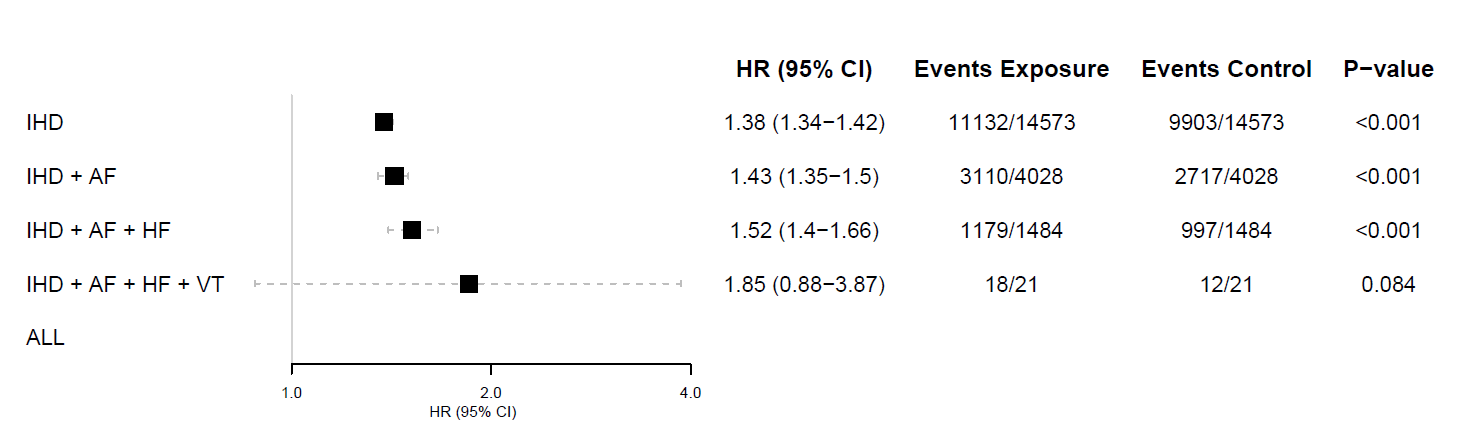


**Figure S1.** Hazard ratios and 95% confidence intervals for the risk of major adverse cardiovascular events over 5-year follow-up in patients with one or multiple newly diagnosed cardiovascular complications versus those who were not newly diagnosed with a cardiovascular complications 4-weeks post intracerebral haemorrhagic stroke.

*AF, atrial fibrillation/flutter; CI, confidence interval; HF, heart failure; ICH, intracerebral haemorrhage; IHD, ischaemic heart disease; MACE, major adverse cardiovascular events; VT, severe ventricular arrhythmia.*

*Hazard ratio (HR), through Cox regression models, reported for propensity-score matched cohort.*


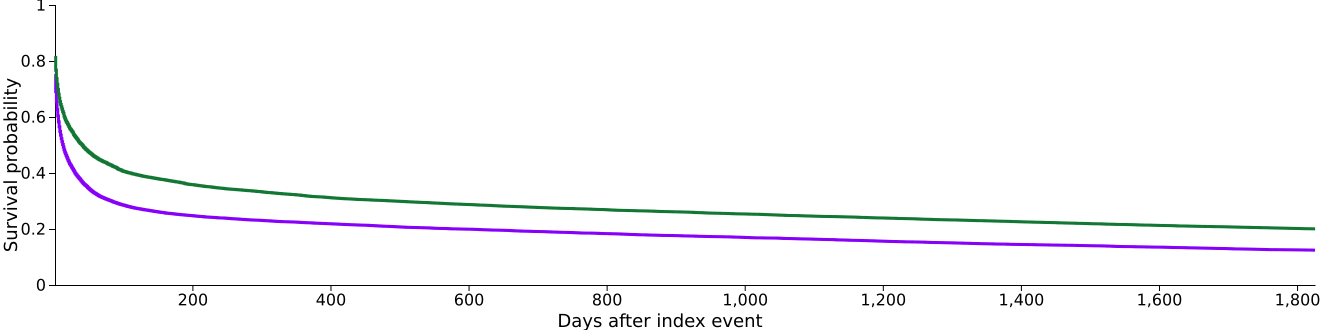


Log-Rank Test, P < 0.01.

**Figure S2**. Kaplan-Meier diagram estimating the probability of the composites of 5-year major adverse cardiovascular events, in daily time intervals. Cumulative major adverse cardiovascular events occurring at 5-year follow up were 12.4% for stroke-heart syndrome cohort (n = 19,343, *green line*), and 20.1% for intracerebral haemorrhage only cohort (n = 17,343, *purple line*). Median days when composites of major adverse cardiovascular events occur for stroke-heart syndrome cohort and intracerebral haemorrhage only cohort were 13 days and 41 days, respectively.

*ICH, intracerebral haemorrhage; MACE, major adverse cardiovascular events; SHS, stroke-heart syndrome*

*
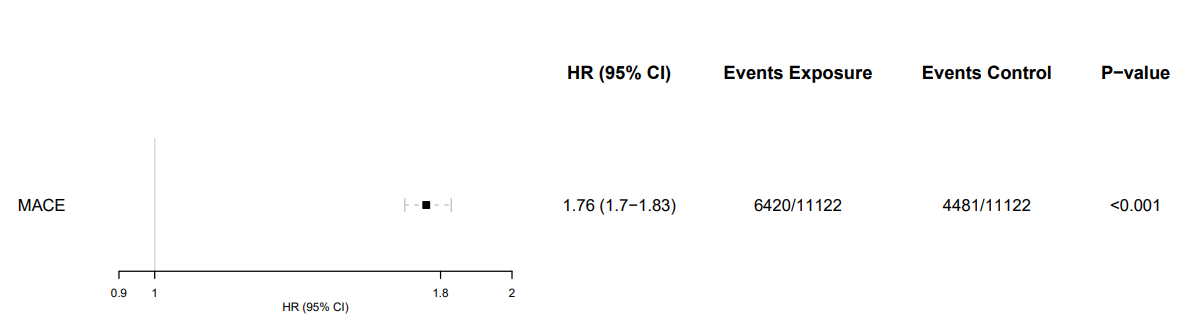
*

**Figure S3.** Hazard ratios and 95% confidence intervals for the risk of major adverse cardiovascular events over 5-year follow-up in stroke-heart syndrome cohort without pre-existing comorbidities (i.e., hypertensive diseases, chronic kidney disease, diabetes mellitus, cerebrovascular diseases, and pulmonary heart disease versus a stroke-heart syndrome cohort without comorbidities prior to intracerebral haemorrhagic stroke.

*CI, confidence interval; ICH, intracerebral haemorrhage; MACE, major adverse cardiovascular events.*

*Hazard ratio (HR), through Cox regression models, reported for propensity-score matched cohort.*
